# Clinical, laboratory, and radiological characteristics of COVID-19-infected children admitted to pediatric intensive care unit: a single-center experience

**DOI:** 10.1186/s43088-021-00168-x

**Published:** 2021-11-14

**Authors:** Heba Mostafa Ahmed, Rehab Muhammad Abd El Kareem, Faten Mohamed Ali, Ahmed Reda Sayed, Yasmen Awadalh Mohamed

**Affiliations:** 1 Department of Pediatrics, Faculty of Medicine, Beni-Suef University, Beni Suef, Egypt; 2 Department of Clinical and Chemical Pathology, Faculty of Medicine, Beni-Suef University, Beni Suef, Egypt; 3 Department of Radiodiagnosis, Faculty of Medicine, Beni-Suef University, Beni Suef, Egypt; 4 Department of Medical Biochemistry and Molecular Biology, Faculty of Medicine, Beni-Suef University, Beni Suef, Egypt

**Keywords:** COVID-19, Pediatric intensive care unit, Multisystem inflammatory syndrome in children

## Abstract

**Background:**

During the second wave of COVID-19, there is an increasing incidence of reported cases in children compared to the early wave. Data on the clinical and laboratory characteristics of COVID-19 in children are evolving, and reports on the characteristics and outcomes of severe COVID-19 in children are still under evaluation. We aimed to describe the clinical, laboratory, and radiological characteristics and outcomes of children with COVID-19 infection admitted to the pediatric intensive care unit (PICU).

**Results:**

The study included 27 children with COVID-19 infection. Fever, respiratory, and gastrointestinal (GIT) symptoms were predominant presenting symptoms in our patients. The median age of our patients was 9 months (2 m–12 years). Comorbidity was reported in 59.3%. The typical laboratory findings were leukocytosis, lymphopenia, elevated C-reactive proteins levels, and elevated d-dimer levels. The most frequent radiological findings were ground-glass opacities in 100% of patients and bilateral findings in 96%, while cardiomegaly was found in 44% of patients. The multisystem inflammatory syndrome was reported in 33% of patients with GIT symptoms were the most frequent presenting symptoms. Myocarditis was reported in 22% of patients. The mortality rate in this cohort was 14.8%. On multivariate analysis, the only predictor of mortality was the development of MIS-C.

**Conclusions:**

COVID-19 is more severe in children with comorbid conditions. Fever, respiratory and gastrointestinal (GIT) symptoms were predominant presenting symptoms. MIS-C is of increasing concern in children with high mortality rates.

**Supplementary Information:**

The online version contains supplementary material available at 10.1186/s43088-021-00168-x.

## Background

Coronaviruses are known pathogens in both animals and humans [1]. At the end of 2019, many cases of viral pneumonia were identified to be caused by a novel coronavirus in Wuhan city in China. The new virus was rapidly spreading, resulting in an epidemic all over China, and then, many cases were reported in other countries all through the world. In February 2020, the World Health Organization (WHO) nominated the disease as coronavirus disease 2019 (COVID-19) [2], and on March 11, 2020, the WHO announced the COVID-19 as a worldwide pandemic [3]. Severe COVID -19 illness in adults is characterized by acute hypoxemia, multi-organ failure, and high mortality [4, 5]. The risk factors for developing severe illness in adults include age, cardiac and respiratory comorbidities, obesity, lymphopenia, and high levels of D-dimer [4, 6]. Initial reports from pediatric studies documented low infection rates and low incidence of pediatric intensive care unit (PICU) admissions [7, 8]. Early reports from China showed that critical illness was reported in only 0.6% of children with COVID-19 [8]. A large PICU report that included 48 children with COVID-19 admitted to 46 North American PICUs between March 14 and April 3, 2020, described these children's clinical characteristics and outcomes and reported a fatality rate of 4.2% [9]. Another large cohort included 632 children with proven COVID-19 infections all through the UK in the period from January 17 and July 3, 2020, showed that 18% (116 children) were admitted to critical care units [10], reflecting the increasing need for critical care management in children with COVID-19. This study aimed to prescribe the clinical characteristics and outcomes of children with COVID-19 admitted to PICU through the period from September 3, 2020, to December 31, 2020.

## Methods

This was a prospective cross-sectional study. All children who presented to the emergency room with acute febrile illness or respiratory symptoms were subjected to full history taking, clinical examination, complete blood picture (CBC), C-reactive protein (CRP), and computed tomography (CT) chest. Suspected COVID-19 infection was based on one of the following: the history of contact with COVID-19-confirmed case or history of contact with a patient with acute febrile illness from communities with confirmed cases, at least 2 of the following clinical features (fever and/or respiratory symptoms, lymphopenia in CBC, characteristic CT findings), severe acute respiratory infection without an obvious cause. Suspected cases underwent either nasopharyngeal swab for PCR or antibody testing (anti-SARS-CV2 IgM and IgG). Positive critically ill patients were admitted to the COVID ward in the PICU, while stable cases were referred to an isolation hospital or home managed. Critically ill cases with negative PCR swap were isolated in the suspected COVID ward in the PICU. The test was repeated after 48 h; if positive, transmitted to the COVID ward and if negative, transmitted to the non-COVID ward in the PICU. The criteria for PICU admission were: oxygen saturation ≤ 90% or Pa O2/FiO2 < 300 at room air, oxygen saturation ≤ 92% or Pa O2/FiO2 < 200 despite oxygen therapy, the presence of septic shock or hemodynamic instability not responding to fluid resuscitation with the need for inotrope support, patients with disturbed consciousness, and if respiratory symptoms were associated with other organs’ failure. Diagnosis of the multisystem inflammatory syndrome in children (MIS-C) was based on the CDC case definition [11], including evidence of COVID-19 infection (positive PCR or rapid test for COVID-19 IgG antibodies). The diagnosis of myocarditis was made based on the presence of left ventricular dysfunction by echocardiography and elevated serum troponin levels. Diagnosis of AKI was based on eGFR < 100 ml/min/1.73m2. eGFR was calculated according to Schwartz formula (eGFR = kL/Scre) where k 0.413, L = patient length or height, Scr = serum creatinine.

Patients were treated according to the Egyptian pediatric consensus recommendations [12] for COVID-19 infection, and MIS-C was treated according to the American Association of Pediatrics recommendations.[13]. For comparison, patients were divided into three groups according to age (age < 1 year, age between 1–5 years, age between 6–12 years).

### PCR for COVID-19 detection

The genesig® Real-Time PCR Coronavirus COVID-19 (CE IVD) is intended to be used to achieve qualitative detection of SARS-CoV-2 viral RNA extracted from nasopharyngeal swabs, oropharyngeal swabs, and sputum from patients. Soon after collection, nasopharyngeal and oropharyngeal secretions, each sample was inactivated by heating at 56 C for 15 min.

Extraction of SARS-CoV-2 viral RNA from nasopharyngeal swabs, oropharyngeal swabs, and sputum was done by Qiagen QIAamp DSP virus spin kit (Qiagen GmbH, lot 163033809) on fully automated QIAcube (Qiagen, Germany). The RNA extraction sample volume of 700 μL from the biological sample was used and re-suspended with the COVID-19 primer/probe tube and the TM StepOne 2X RT-qPCR Master Mix then gently swirled to mix. The thermocycler was programmed with an initial cycle of 55 °C for 10 min, then 95 °C for 2 min and 45 cycles of 95 C for 10 s and 60 C for 60 s. Reverse transcription 10 min 55 °C one cycle using TM StepOne (Applied Biosystem, Thermo Fisher Scientific).

### Detection of COVID-19 antibodies

COVID-19 antibodies were detected using Elecsys Anti-SARA-COV-2 commercial kits (Roche diagnostic GmbH, Germany) on Cobas e 411 (Roche, Hitachi, Germany).

### Measurement of D-dimer and CRP

Both tests were measured using the nephelometric assay that utilizes antibody-coated latex particles. Both tests were done on MISPA_i 2, specific protein analyzer (AGAPPE Diagnostics Switzerland GmbH).

### Reporting of CT findings

#### CORADS grading

CORADS-1; COVID-19 is highly unremarkable, CT is normal or the findings seen are indicative of non-infectious disease, CORADS-2; suspicion level of COVID-19 infection or the findings may suggest other infections; CORADS-3; indeterminate findings of COVID-19 infection, and the Ct findings include findings of infection and unsure if COVID-19 infection is present, CORAD-4; high suspicious for COVID-19 infection but not typical as being unilateral ground-glass opacity, confluent or being multifocal consolidative areas with atypical location, and CORADS-5; very high suspicion level of COVID-19 infection with typical CT findings [14].

#### Semiquantitative scoring system

The CT-SS was calculated based on the lobar involvement extent. Each of the five lung lobes was visually scored using a scale of 0–5, with 0 indicates no remarkable involvement, 1 indicates less than 5% lobar involvement, 2 indicates 5–25% lobar involvement, 3 indicates 26–49% lobar involvement, 4 indicates 50–75% lobar involvement, and 5 indicates more than 75% lobar involvement. The total CT score is calculated as the sum of the individual lobar scores ranging from 0 (no remarkable involvement) to 25 (maximum involvement). [15, 16] and defined as: 0, none; 1 to 5, minimal; 6 to 10, mild; 11 to 15, moderate; and ≥ 16, severe involvement of the lungs [17].

### Statistical analysis

The SPSS software (Statistical Package for the Social Sciences, version 22.0, SPSS Inc, Chicago, IL, USA) was used for analysis. Qualitative data were compared using the Chi-square test, and values were expressed as numbers and percentages. Parametric quantitative independent groups were examined using the t-test, or the Mann–Whitney test, and values were expressed as mean ± standard deviation. One-way ANOVA test was used to compare continuous variables between the three age groups. Predictors for poor outcomes were studied using the binary logistic regression test. P-value was set at less than 0.05 for significant results.

## Results

Out of 36 patients with suspected COVID-19 admitted to the PICU, 27 were proven positive for COVID-19, including 16 (59%) males and 11 (40.7%) females. The median age of our study group was 9 months (2 months–12 years). The patients were categorized according to their ages into three groups: G1 age less than one year (17; 63%), G2: 1–5 years (3;11%), and group 6–12 years (7; 26%). The median duration of pre-admission illness was four days (1–12 days). History of contact to COVID-19-positive patients was documented in 19 (70.4%) patients. History of comorbid chronic disease was documented in 16 (59.3%) of the patients (4 patients with congenital heart disease, one patient with bronchial asthma, 4 with central nervous system (CNS) disorders, 2 with chronic hematological disorders, 3 patients with nephrotic syndrome, one patient with growth hormone deficiency, and one patient with brain tumor). The presenting symptoms were fever (23; 85.2%), cough 18 (66.7%), shortness of breath (15; 55.5%), gastrointestinal tract (GIT) symptoms (12; 44.4%), convulsions(7;25.9%), altered sensorium (11;59.3%), shock (10;37%). MIS-C was reported in 9 (33.3%) of patients (Additional file 1: Digital Component 1). Nasal CPAP was indicated in 3 patients, while 6 (22.2%) required invasive mechanical ventilation. The laboratory characteristics, complications, and medications received by the whole study group are shown in Table 1. High CRP > 5 mg/dl and ferritin levels > 200 mg/dl were detected in 100% of patients and elevated d-dimer more than 500 ng/mL in 63% of the patients. Anemia, leukocytosis, and lymphopenia were frequently observed. AKI was documented in 10 patients; 2 of them required renal replacement therapy. The radiological findings were ground-glass opacities in 49%, bilateral findings in 30%, cardiomegaly in 44%. The median duration of PICU admission was 14 days (8–20 days). Echocardiographic findings included mild left ventricular dysfunction in 1 patient and moderate-to-severe left ventricular dysfunction in 5 patients; one of them had left coronary artery dilation and presented clinically with cardiogenic shock. As regarding treatments received by the patients, corticosteroids were given to 22 (81.5%) patients. Eleven patients received pulse methylprednisolone (9 patients with MIS-C and 2 patients with myocarditis not fulfilling cafeteria for MIS-C), while 11 patients received oral prednisolone. IVIG was given to 3 patients with severe MIS-C, and one patient with severe COVID-19 and shock. Low molecular weight heparin was given to 14 patients with high d-dimer, while antiplatelets were given to 17 patients. Comparison between the three age groups is shown in Table 2 with no significant differences between the three groups except for associated comorbidity, which was significantly higher in groups aged 1–12 than in patients younger than one year of age. At the same time, the frequency of cardiomegaly and myocarditis was significantly higher in patients younger than six years in comparison with older children. Comparisons between patients with and without MIS-C are shown in Table 3, Additional file 1: Digital Component 2. The frequency of GIT symptoms, shock at presentation, thrombocytopenia, lymphopenia, AKI, and elevated d-dimer levels were significantly higher in MIS-C patients. On logistic regression analysis, only AKI was an independent predictor for MIS-C (odds ratio; 2.35, SE; 1.14. 95% CI; 1.12–98.92).

**Table 1:** Clinical and laboratory characteristics of the study group (27 patients)

	Frequency	Percent
Laboratory data		
CBC parameters		
Anemia	17	63.00
Thrombocytopenia < 150 × 10^3^	6	22.20
Leukocytosis	16	59.30
Lymphopenia < 1000/ccm^3^	14	51.90
Hypernatremia > 45 mEq/L	14	51.90
K disturbances		
Low	6	22.20
High	10	37.00
Metabolic acidosis (ph < 7.2)	12	44.40
High CRP > 5 mg/dl	27	100.00
High ferritin > 200 mg/dl)	27	100.00
High d- dimer > 500 mg/dl	17	63.00
Coagulopathy (INR > 1.2)	13	48.10
High troponin I (> 100 pg/mI)	6	22.20
Complications		
Pneumonia	18	66.70
ARDS	5	18.50
AKI (eGFR < 60 ml/min/m^2^)	10	37.00
Myocarditis	6	22.20
Medications		
Vasoactive infusion	8	29.60
Antibiotics	26	96.30
Corticosteroids	22	81.50
Anticoagulants	14	51.80
Antiplatelets	17	63.00
Remdesivir	1	4.00
IVIG	4	14.80
Outcomes		
Discharge	23	85.20
Mortality	4	14.80

AKI, acute kidney injury; ARDS, acute respiratory distress syndrome; CBC, complete blood counts; CRP, C-reactive protein; eGFR, estimated glomerular filtration rate; INR, international normalization ratio; IVIG, intravenous immunoglobulins; K, potassium

**Table 2:** Comparison between the three age groups

	Age group			p
	< 1 y (n = 17)	1–5 y (n = 3)	6–12 y (n = 7)	
**Demographic and clinical characteristics**				
Male sex	10 (58.80%)	2 (66.70%)	4 (57.10%)	.960
Contact to a positive case	10 (58.80%)	2 (66.70%)	7 (100%)	.132
Comorbidity	7 (41.20%)	3 (100%)	6 (85.70%)	.041
Fever	13 (76.50%)	3 (100%)	7 (100%)	.251
Cough	7 (41.20%)	2 (66.70%)	5 (71.40%)	.446
Dyspnea	6 (35.30%)	3 (100%)	6 (85.70%)	.677
GIT symptoms	9 (52.90%)	1 (33.30%)	2 (28.60%)	.506
DCL	8 (47.10%)	2 (66.70%)	1 (14.30%)	.207
Convulsions	4 (23.50%)	2 (66.70%)	1 (14.30%)	.208
Shock	8 (47.10%)	1 (33.30%)	1 (14.30%)	.316
**Laboratory characteristics**				
Anemia	11 (64.70%)	2 (66.67%)	4 (57.14%)	.932
Thrombocytopenia	5 (29.4%)	0 (0.00%)	1 (14.3%)	.445
Leukocytosis	8 (47.1%)	3 (100%)	5 (71.4%)	.170
Hypernatremia	8 (47.1%)	2 (66.7%)	4 (57.1%)	.779
Hypokalemia	4 (23.5%)	1 (33.3%)	1 (14.3%)	.466
Hyperkalemia	8 (47.1%)	0 (0.00%)	2 (28.6%)	
Hypoxemia	7 (41.2%)	2 (66.7%)	3 (42.9%)	.712
Coagulopathy	7 (41.2%)	3 (100%)	3 (42.9%)	.162
High d-dimer	10 (58.8%)	3 (100%)	4 (57.1%)	.369
High troponin I	3 (17.65%)	1 (33.33%	2 (28.6%)	.87
**Complications**				
AKI	7 (41.2%)	1 (33.3%)	2 (28.6%)	.836
Myocarditis	4 (23.5%)	1 (33.3%)	1 (14.3%)	.020
Pneumonia	8 (47.1%)	3 (100%)	7 (100%)	.284
MIS-C	5 (29.4%)	2 (66.7%)	2 (28.6%)	.430
**CT findings**				
CORADS				
3.00	4 (23.5%)	0 (0.0%)	0 (0.0%)	.596
4.00	9 (52.9%)	2 (66.7%)	5 (71.4%)	
5.00	4 (23.5%)	1 (33.3%)	2 (28.6%)	
Pneumonia	4 (23.5%)	2 (66.7%)	3 (42.9%)	.284
Bilateral findings	5 (29.4%)	1 (33.3%)	2 (28.6%)	.227
Cardiomegaly	11 (64.7%)	0 (0.0%)	1 (14.3%)	.02

AKI, acute kidney injury; GIT, gastrointestinal tract; MIS-c, multisystem inflammatory syndrome in children; CORADS, COVID-19 reporting and data system

**Table 3:** Comparison between patients with and without MIS-C

	MIS-C		p-value
	No	Yes	
*Clinical data*			
Male sex	11 (61.1%)	5 (55.6%)	.78
Comorbidity	13 (72.2%)	3 (33.3%)	.05
GIT	5 (27.8%)	7 (77.8%)	.014
Shock	4 (22.2%)	6 (66.7%)	.024
Myocarditis	2 (11.1%)	4 (44.4%)	.05
IMV	2 (11.1%)	4 (44.4%)	.05
Mortality	1 (5.6%)	3 (33.3%)	.17
*Laboratory data*			
Anemia	10 (55.6%	7 (77.8%)	.26
Thrombocytopenia	1 (5.6%)	5 (55.6%)	.003
Leukocytosis	10 (55.6%)	6 (66.7%)	.58
Lymphopenia	6 (33.3%)	8 (88.9%)	.006
AKI	3 (16.7%)	7 (77.8%)	.002
Coagulopathy	7 (38.9%)	6 (66.7%)	.17
High d-dimer	8 (44.4%)	9 (100.0%)	.005
High troponin I	2 (11.1%)	4 (44.4%)	.91

AKI, acute kidney injury; GIT, gastrointestinal tract; IMV, invasive mechanical ventilation; MIS-c, multisystem inflammatory syndrome

The mortality rate in our cohort was 14.8% (4 patients, 3 of them had MIS-C, and 3 had chronic comorbidity). On logistic regression analysis, the development of MIS-C was a significant predictor of mortality (Table 4).

**Table 4:** Binary logistic regression analysis for predictors of mortality

Variable	Odds ratio	SE	95% CI	p-value
Respiratory distress	1.25	1.01	.17–9.02	.831
GIT symptoms	.012	1.01	.11–5.77	.824
Shock	1.17	1.02	.16–8.52	.879
AKI	1.17	1.02	0.16–8.52	..879
D-dimer > 1000 ng/ml	1.6	1.23	.14–18.01	.751
Myocarditis	.48	.85	.09- 2.55	.395
MIS-C	13.6	1.3	1.23–78.05	.034

AKI, acute kidney injury; GIT, gastrointestinal tract; IMV, invasive mechanical ventilation; K, potassium; MIS-c, multisystem inflammatory syndrome in children

## Discussion

Most of the data on critically ill COVID-19-infected children comes from developed countries; the clinical, laboratory, and radiological findings in children admitted to PICU with COVID-19 infection are described here for a better understanding of the underlying risk factors that can predict the outcome of children from a resource-constrained developing country. Most of our patients were younger than one year of age in our cohort, while only 11% of patients were in the group between 1 and 5 years of age. In line with our findings, Swann et al. [10] reported that the most common age group was younger than one year, and only 12.9 percent of their PICU-admitted patients were in the age group between 1 and 4 years. Comorbid conditions were reported in 59.3% of our patients; most of them were older than one year of age. Similar results were observed in the study by Swann et al. [10], who mentioned that 54.3% of their cohort had comorbidities. In comparison, Shekerdemian and her peers [9] reported a higher incidence of comorbidity among their patients (83%), similar to that reported in adults [4–6]. Most of our cases had fever at presentation similar to what was reported in other studies [9, 10]. In our cohort, fever and GIT symptoms were more prevalent in children younger than one year, while older age groups reported respiratory symptoms. In the study of Swann et al. [10], diarrhea and vomiting were reported in 34.5% and 39.7%, respectively. Similarly, González-Dambrauskas et al. [18] reported that 35% of their patients presented GIT symptoms. Contrary to our results, in the study by Shekerdemian et al. [9], only one patient out of 48 presented GIT symptoms. The research time frames could still explain this discrepancy in the presenting symptoms. In contrast, the study with a higher incidence of respiratory symptoms was conducted out at an earlier time in 2020 year. Our study and the study by González-Dambrauskas et al. [18] were conducted during the 2nd half of 2020, indicating the changing nature of the virus during the first and second waves. In our study, IMV was indicated in 22.2% of patients. However, in the study by Shekerdemian et al. [9] and the study by González-Dambrauskas et al. [18], higher incidences of IMV were reported (38% and 47%, respectively). Compared to these two studies, the lower rate of IMV in our cohort may be due to the lower rates of severe respiratory symptoms and ARDS. In our study, 33% of our patients met the CDC criteria of MIS-C, similar to what was reported by Swann et al. [10], where 32.7% (38/116) of COVID-19-positive children admitted to the PICU met the WHO criteria for MIS-C. In contrast to the study by Swann et al. [10], which reported higher rates of MIS-C in the older age group, there were no significant age differences in patients with and without MIS-C. Our explanation for this difference could be due to the different ethnic groups, including older children (up to 19 years of age). In patients with MIS-C, GIT symptoms predominated significantly compared to non-MIS-C patients, similar to those reported in other studies [10, 19]. In addition, platelet counts and lymphocytic counts were significantly lower in MIS-C patients. Other researchers have reported similar findings [10, 19]. Serum creatinine and d-dimer levels were also found to be significantly higher in patients with MIS-C. In the study by Swann et al. [10], higher creatinine levels were reported in patients with MIS-C. However, they did not comment on differences in d-dimer levels in both groups. Pereira et al. [20] reported that d-dimer levels of more than 1000 ng/dl were significantly associated with MIS-C (p-value; 007). In the current study, we reported a mortality rate of 14.8%, which was higher than that reported in other studies. In the study conducted by Swann et al. [10], a 0.9% (6/651) mortality rate was reported for a cohort of patients admitted to hospital and PICU wards. However, the mortality rate of PICU-admitted patients was not reported. Similarly, in the study of Shekerdemian et al. [9], the mortality rate was 4%. However, at the time of submission, 19% of their cohort were still admitted with critical illnesses, so the total cohort's actual mortality could be understated. In our cohort, one of the four patients died had died only after 5 h of admission ( a case of nephrotic syndrome on treatment with mycophenolate mofetil complained of high-grade fever, abdominal pain, vomiting, diarrhea, and skin rash received home treatment without improvement of her complaints and presented to our ER after 8 days with circulatory shock and severe myocardial dysfunction necessitated IMV and inotropes support, but unfortunately she passed out and her swap revealed COVID-19 positive). Two of the remaining three patients had severe cerebral palsy and impaired respiratory function, while only one was previously healthy before admission. The presence of MIS-C was a significant predictor of mortality in this study. In the study by Pereira et al. [20], hypoxemia and MIS-C were associated with mortality. In our study, we found no association between the findings of the CT and the risk of MIS-C or mortality despite previous reports of this association [15, 17, 21]. This may be related to the predominance of GIT symptoms over respiratory symptoms, particularly among MIS-C patients. Although only one patient received antiviral therapy due to our limited resources, the outcome of our patients did not differ greatly from the other reports, as the most serious cases are those with MIS-C that may benefit from immunosuppressants than antiviral therapy.

Study strength: this study describes clinical, laboratory, and radiographic data of children admitted to PICU due to COVID-19 infection in a prospective study that is not limited to patient records and provides insight into differences in clinical settings of the disease and outcomes in a different ethnicity with limited resources.

Study limitations: small sample size as the disease is much milder in children than adults, and this study represents a single center's experience.

## Conclusions

COVID-19 is more severe in children with comorbid conditions. Fever, respiratory, and gastrointestinal (GIT) symptoms were predominant presenting symptoms. MIS-C is of increasing concern in children with high mortality rates.

## Supplementary Information

**Additional file 1**. Digital Component 1 and Digital Component 2.

## Data Availability

Not applicable.

## Abbreviations

AKI: Acute kidney injury
CBC: Complete blood picture
CDC: Centers for Disease Control and Prevention
CRP: C-reactive protein
CT: Computed tomography
GIT: Gastrointestinal tract
MIS-C: Multisystem inflammatory syndrome in children
PICU: Pediatric intensive care unit
WHO: World Health Organization
