# Supplementary material for: Clinical, laboratory, and radiological characteristics of COVID-19-infected children admitted to pediatric intensive care unit: a single-center experience
Source: Beni Suef Univ J Basic Appl Sci. 2021 Nov 14;10(1):79. doi: 10.1186/s43088-021-00168-x (PMC8590861; doi:10.1186/s43088-021-00168-x)
Supplement: Supplementary file 1 — Additional file 1. Digital Component 1 and Digital Component 2. [file 43088_2021_168_MOESM1_ESM.docx]

Supplemental digital component 1: Characteristic of patients with MIS-C

| Case No. | 1 | 2 | 3 | 4 | 5 | 6 | 7 | 8 | 9 |
| --- | --- | --- | --- | --- | --- | --- | --- | --- | --- |
| Age | 6.5y | 8m | 4m | 8m | 1.2y | 4m | 12y | 2.5y | 3m |
| Sex | M | M | F | F | F | M | F | M | F |
| Comorbidity | - | - | - | - | CP | - | NS | Brain tumor | - |
| Fever | + | + | + | + | + | + | + | + | + |
| Cough | + | - | - | - | - | - | + | + | + |
| RD | + | + | + | + | + | + | - | + | + |
| Diarrhea | + | + | + | + | + | + | + | - | + |
| Vomiting | + | + | + | + | + | + | + | - | - |
| DCL | + | + | + | + | + | + | + | + | - |
| Shock | + | + | + | + | + | + | + | - | - |
| Convulsions | - | + | - | - | + | - | - | + | - |
| IMV | - | + | - | - | - | + | + | + | - |
| Myocarditis | - | + | + | - | - | + | + | - | - |
| Steroids | + | + | + | + | + | + | + | + | + |
| Anti-coagulants | + | + | + | + | + | + | + | + | + |
| IVIG | - | + | + | - | - | + | - | - | - |
| Hemoglobin (gm%) | 8.3 | 8 | 6.8 | 10 | 14.2 | 8.1 | 8 | 8.4 | 8.3 |
| Platelets x10^3^/cm^3^ | 200 | 91 | 132 | 200 | 320 | 141 | 130 | 253 | 137 |
| Leucocytes x10^3^/cm^3^ | 26 | 4 | 26 | 13 | 18 | 47 | 20 | 25 | 10.4 |
| Lymphocytes/ cm^3^ | 860 | 680 | 420 | 1000 | 340 | 520 | 1000 | 700 | 1080 |
| Sodium (mEq/dl) | 150 | 156 | 133 | 152 | 152 | 146 | 146 | 148 | 142 |
| Potassium (mEq/dl) | 6.5 | 2.8 | 6.7 | 4 | 3 | 2.6 | 5.6 | 4.6 | 5.4 |
| Creatinine (mg/dl) | 2.6 | 1 | 2.4 | 1.3 | 3.2 | 3.6 | 1.3 | 0.9 | 0.7 |
| INR | 2 | 1.7 | 1.3 | 1.2 | 3.2 | 1.3 | 1.2 | 1.9 | 1 |
| CRP (mg/dl) | 30 | 35 | 35 | 24 | 16.5 | 96 | 24 | 285 | 24 |
| Ferritin (mg/dl) | 780 | 925 | 2714 | 900 | 597 | 2000 | 1000 | 922 | 448 |
| D-dimer (ng/ml) | 1000 | 4000 | 1000 | 600 | 1500 | 4600 | 700 | 800 | 6.8 |
| CORADS | 5 | 5 | 4 | 4 | 4 | 4 | 5 | 4 | 4 |
| Severity score | 5 | 11 | 5 | 3 | 4 | 8 | 10 | 7 | 10 |
| GGO | - | - | + | - | - | - | + | + | - |
| Pneumonia | - | - | - | + | + | - | + | + | - |
| Bilateral findings | - | - | + | - | - | - | + | - | - |
| Cardiomegaly | - | + | + | + | - | + | + | - | - |
| Duration of admission (day) | 10 | 16 | 14 | - | 12 | 14 | 0 | 12 | 10 |
| Mortality | - | + | - | - | + | - | + | - | - |

AKI; acute kidney injury, ARDS; acute respiratory distress syndrome, CBC; complete blood counts, CORADS; COVID-19 reporting and data system, CRP; C-reactive protein, DCL; disturbed conscious level , eGFR; estimated glomerular filtration rate, GGO; ground glass opacities, INR; international normalization ratio, IVIG; intravenous immunoglobulins , RD; respiratory distress,

Supplemental digital component 2: Comparison between patients with and without MIS-C

| **Patients** | | | |
| --- | --- | --- | --- |
|  | With MIS-C (n=9) | Without MIS-C (n=18) | p |
| Age | 2.696±4.017 | 3.246± 4.467 | . 753 |
| Hemoglobin (gm/dl) | 8.900±2.148 | 9.383± 1.303 | .472 |
| Platelets x10^3^ /ccm | 178.222± 59.156 | 329.777± 148.189 | .007 |
| TLC x10^3^ /ccm | 21.044±12.335 | 17.850± 6.195 | .374 |
| Lymphocytes x10^3^ /ccm | 633.333± 320.312 | 1128.944± 280.131 | .000 |
| Sodium (mEq/L) | 147.222± 6.741 | 141.777± 5.966 | .042 |
| Potassium (mEq/L) | 4.577± 1.573 | 4.438± .861 | .768 |
| Creatinine (mg/dl) | 1.888± 1.077 | .600± .391 | .000 |
| INR | 1.644±.676 | 1.486± .761 | .602 |
| CRP (mg/dl) | 63.277± 86.425 | 43.722± 45.875 | .446 |
| Ferritin (mg/dl) | 1142.888± 732.398 | 662.388± 557.797 | .069 |
| D-dimer (ng/ml) | 2.333± 2.239 | .538± .209 | .002 |
| Severity score | 9.222± 4.816 | 8.611± 5.203 | .771 |
| Duration of admission (days) | 13.111± 3.333 | 13.0556± 3.455 | .969 |

CRP; C-reactive protein, INR; international normalization ratio, TLC; total leukocyte counts.
